# Clinical Outcomes and Safety Profile of Camrelizumab Plus Famitinib in Persistent, Recurrent, or Metastatic Cervical Cancer

**DOI:** 10.32604/or.2026.088774

**Published:** 2026-10-28

**Authors:** Xiaojing Liu, Beibei Hua, Yang Xia, Huijie Huang, Areai Hakewati, Zeze Jin, Tumaris Halifu, Zaihua Xu

**Affiliations:** Department of Radiation Oncology, The Friendship Hospital of Ili Kazakh Autonomous Prefecture, 58 Chongqing Road, Yining, Xinjiang Uygur Autonomous Region, China

**Keywords:** Cervical cancer, camrelizumab, famitinib, progression-free survival, disease control rate, safety profile

## Abstract

**Background:** Comparative real-world evidence for camrelizumab plus famitinib versus PD-1 inhibitor plus platinum- and/or taxane-based chemotherapy with or without bevacizumab in persistent, recurrent, or metastatic cervical cancer remains limited. This study evaluated the clinical outcomes and safety of these strategies. **Methods:** This single-center retrospective cohort study included 111 patients treated between July 2023 and October 2025; 42 received camrelizumab plus famitinib and 69 received the control regimen. Progression-free survival (PFS) was the primary endpoint. Overall survival (OS), tumor response, treatment-related adverse events (TRAEs), propensity score-based robustness analyses, and exploratory post-baseline marker analyses were also assessed. **Results:** Full-cohort and propensity score-matched Kaplan-Meier analyses showed longer PFS with camrelizumab plus famitinib (*p* = 0.026 and *p* = 0.004, respectively). Treatment-effect estimates also favored camrelizumab plus famitinib in unadjusted Cox (*p* = 0.028), residual imbalance-adjusted Cox (*p* = 0.041), 1:1 propensity score-matched Cox (*p* = 0.010), trimmed inverse probability of treatment weighting Cox (*p* = 0.006), and overlap-weighted Cox analyses (*p* = 0.005), whereas the prespecified baseline-adjusted Cox model was not statistically significant (*p* = 0.062). Disease control rate was higher (*p* = 0.038), grade 3–5 myelosuppression was lower (*p* = 0.010), and hypertension was higher (*p* = 0.023) with camrelizumab plus famitinib. OS did not differ significantly. Liver metastasis (*p* = 0.001) and higher baseline neutrophil-to-lymphocyte ratio (*p* = 0.037) were associated with shorter PFS. **Conclusions:** Camrelizumab plus famitinib showed a favorable PFS signal across several analyses, higher disease control, and a distinct safety profile, while the primary baseline-adjusted estimate and OS remained statistically non-significant.

## 1 Introduction

Cervical cancer remains one of the most common gynecologic malignancies worldwide and continues to impose a substantial disease burden, particularly among patients diagnosed at an advanced stage or experiencing relapse after definitive therapy [1]. Persistent, recurrent, or metastatic cervical cancer represents a clinically heterogeneous population with limited curative options, and systemic therapy is the main treatment approach for most patients in this setting [2].

Platinum-based chemotherapy combined with a taxane has been widely used as a systemic treatment backbone for recurrent or metastatic cervical cancer [3]. The addition of bevacizumab to chemotherapy further established antiangiogenic therapy as an important component of treatment for selected patients [4]. More recently, immune checkpoint inhibitors targeting programmed cell death protein 1 (PD-1) or programmed death ligand 1 (PD-L1) have been incorporated into combination regimens with platinum-based chemotherapy with or without bevacizumab, and several phase III studies have supported the role of immunotherapy-based systemic treatment in advanced cervical cancer [5,6,7]. These therapeutic advances have expanded available treatment options but have also increased the need to define the clinical outcomes and safety profiles of different combination strategies in routine practice [8].

Camrelizumab is a PD-1 inhibitor that has shown antitumor activity across multiple solid tumors and has been explored in immunotherapy-based combinations for gynecologic malignancies [9]. Famitinib is an oral multi-target tyrosine kinase inhibitor (TKI) with inhibitory activity against angiogenesis-related pathways, including vascular endothelial growth factor receptors (VEGFRs), platelet-derived growth factor receptors (PDGFRs), and KIT [10]. The combination of immune checkpoint blockade and angiogenesis inhibition has a biological rationale because tumor angiogenesis is closely linked to immune suppression, impaired immune-cell trafficking, and an immunologically unfavorable tumor microenvironment [11,12].

Despite this rationale, clinical evidence on camrelizumab plus famitinib in persistent, recurrent, or metastatic cervical cancer remains limited. In current practice, PD-1 inhibitor plus platinum- and/or taxane-based chemotherapy with or without bevacizumab is a commonly used comparator regimen, but comparative data addressing camrelizumab plus famitinib against this treatment pattern are insufficient. A comprehensive evaluation should include not only survival outcomes, such as progression-free survival (PFS) and overall survival (OS), but also tumor response, treatment-related adverse events (TRAEs), and clinically accessible markers that may characterize heterogeneity in benefit and tolerance [13,14,15].

Therefore, this study aimed to evaluate the clinical outcomes and safety profile of camrelizumab plus famitinib in patients with persistent, recurrent, or metastatic cervical cancer, compared with PD-1 inhibitor plus platinum- and/or taxane-based chemotherapy with or without bevacizumab, using survival, response, safety, propensity score-based robustness, and exploratory clinical marker analyses.

## 2 Materials and Methods

### 2.1 General Information

This single-center retrospective cohort study included patients with persistent, recurrent, or *de novo* metastatic cervical cancer who received systemic treatment between 1 July 2023 and 31 October 2025. Follow-up data were updated through 10 February 2026. The study compared camrelizumab plus famitinib with a control regimen consisting of a PD-1 inhibitor plus platinum- and/or taxane-based chemotherapy with or without bevacizumab. Consecutive patients were screened from electronic medical records, treatment records, laboratory reports, imaging reports, and follow-up documentation. Among 127 screened patients, 16 were excluded after eligibility assessment, and 111 patients were included in the final cohort. Of these, 42 patients received camrelizumab plus famitinib and 69 patients received the control regimen. The full cohort was used for efficacy and safety analyses, and 34 matched pairs were retained after propensity score matching. This study was conducted in accordance with the Declaration of Helsinki and was approved by the Science and Technology Ethics Committee of The Friendship Hospital of Ili Kazakh Autonomous Prefecture (approval No. KYSB-2025-72; approved 28 March 2025). The period from 1 July 2023 to 31 October 2025 refers to the clinical treatment eligibility window rather than the initiation of research-specific data collection; retrospective screening and extraction of study data were undertaken after ethics approval. The requirement for written informed consent was waived because of the retrospective design and de-identified data analysis.

### 2.2 Patient Criteria

Patients were eligible if they met the following criteria: histologically confirmed cervical cancer; age 18 to 75 years; persistent, recurrent, or *de novo* metastatic disease; receipt of at least one administration of camrelizumab plus famitinib or a PD-1 inhibitor plus platinum- and/or taxane-based chemotherapy with or without bevacizumab; and available clinical, laboratory, imaging, safety, and follow-up records sufficient for efficacy and safety assessment. Disease status was determined at the index date and classified into mutually exclusive categories. Persistent disease was defined as residual cervical cancer that remained detectable after completion of initial definitive treatment without a documented complete response; recurrent disease was defined as newly detected locoregional or distant disease after a documented complete response following prior definitive treatment; and *de novo* metastatic disease was defined as distant metastatic disease present at the initial diagnosis before initiation of systemic treatment for advanced disease. Distant metastases that developed after a previous complete response were classified as recurrent disease rather than *de novo* metastatic disease. Patients who discontinued treatment before completion of two cycles because of documented disease progression, death, or intolerable toxicity remained eligible provided that the index treatment date and relevant outcome information were clearly documented. No patient was excluded solely because progression, death, or treatment-limiting toxicity occurred before completion of two treatment cycles.

Intolerable toxicity was defined as a treatment-related adverse event that remained clinically unacceptable despite appropriate treatment interruption, supportive care, and dose adjustment of modifiable agents and resulted in permanent discontinuation of the index regimen. This category included severe toxicities and persistent or recurrent clinically significant lower-grade toxicities when permanent discontinuation was documented after these measures; toxicity severity was graded according to the Common Terminology Criteria for Adverse Events version 5.0. Patients were excluded if they had another active malignancy, cervical involvement from a non-cervical primary tumor, treatment with a markedly heterogeneous non-study systemic regimen as the main concurrent therapy, uncontrolled autoimmune disease requiring long-term systemic corticosteroids, uncontrolled infection, severe cardiovascular or cerebrovascular events, severe hepatic or renal failure before treatment, unavailable baseline imaging, missing key baseline or outcome information, only one treatment administration without evaluable efficacy, safety, or survival records, or unclear treatment initiation or progression dates.

### 2.3 Treatment

Patients were classified according to the index systemic regimen actually initiated, and the index date was defined as the first administration date of that regimen. Patients in the camrelizumab plus famitinib group received camrelizumab 200 mg by intravenous infusion on day 1 of each 21-day cycle and famitinib 20 mg orally once daily continuously during each cycle. Camrelizumab was administered every 3 weeks without dose reduction; temporary interruption was permitted for treatment-related toxicity. Famitinib could be temporarily interrupted or dose-reduced for toxicity. Treatment was continued until radiographic or unequivocal clinical disease progression, intolerable toxicity, initiation of another systemic anticancer regimen, death, or treatment discontinuation decided by the patient or treating physician. Patients in the control group received a PD-1 inhibitor in combination with platinum- and/or taxane-based chemotherapy, with or without bevacizumab. The PD-1 inhibitors used included pembrolizumab 200 mg, sintilimab 200 mg, or tislelizumab 200 mg, administered intravenously on day 1 of each 21-day cycle. The chemotherapy component comprised cisplatin 50 mg/m^2^ intravenously on day 1, carboplatin at an area under the concentration-time curve of 5 mg·min/mL intravenously on day 1, or nedaplatin 80 mg/m^2^ intravenously on day 1, and/or paclitaxel 175 mg/m^2^ intravenously on day 1, nab-paclitaxel 175 mg/m^2^ intravenously on day 1, or docetaxel 75 mg/m^2^ intravenously on day 1. Cytotoxic chemotherapy was administered on a 21-day cycle for 4–6 cycles unless earlier discontinuation was required because of disease progression or intolerable toxicity. When bevacizumab was used, it was administered intravenously at 15 mg/kg on day 1 every 3 weeks and could be continued after completion of cytotoxic chemotherapy in patients without progression or intolerable toxicity. The decision to add bevacizumab was based on the treating physician’s assessment of clinical suitability, taking into account prior treatment history, contraindications to antiangiogenic therapy, treatment tolerance, and the patient’s overall clinical condition.

Treatment allocation was not determined by the study protocol or a prespecified biomarker-based stratification algorithm; the regimen received reflected routine clinical decision-making. PD-L1 combined positive score (CPS) was not available for all patients and was not used as an inclusion criterion or mandatory treatment-stratification variable. Treatment line and prior treatment history were recorded as pretreatment variables. To address confounding by indication, the propensity score model included age, Eastern Cooperative Oncology Group performance status (ECOG PS), current treatment line, initial International Federation of Gynecology and Obstetrics (FIGO) stage, and disease status; the primary progression-free survival (PFS) model included treatment group, liver metastasis, baseline neutrophil-to-lymphocyte ratio (NLR), and recurrence field, and the residual imbalance-adjusted sensitivity model additionally included hemoglobin (Hb) and prior PD-1/PD-L1 therapy. Treatment-related variables included current treatment line, platinum agent, taxane agent, bevacizumab use, and local therapy during treatment. Current treatment line was classified as first-line, second-line, or third-line or later. Bevacizumab use and local therapy during treatment were recorded as binary variables, and dose modification or interruption and treatment discontinuation were extracted from treatment documentation. Platinum-agent, taxane-agent, and bevacizumab variables reflected the actual systemic treatment exposures documented during the index treatment period. These variables were reported descriptively and did not redefine the primary treatment-group classification.

### 2.4 Response Evaluation and Follow-Up

Baseline radiologic assessment was performed within 28 days before the index date. Contrast-enhanced computed tomography of the chest, abdomen, and pelvis was the principal imaging modality, with pelvic magnetic resonance imaging used when required for more accurate assessment of locoregional disease. The same imaging modality was used for serial assessment whenever possible. The first post-baseline tumor assessment was performed after completion of two treatment cycles, with the exact assessment date reflecting routine clinical scheduling. Subsequent assessments were generally performed every 6–9 weeks thereafter, and imaging obtained earlier because of suspected disease progression was also included. Tumor response was retrospectively extracted according to Response Evaluation Criteria in Solid Tumors (RECIST) version 1.1 from official radiology reports and oncologic response assessments documented in the medical record. No blinded independent central radiologic review was performed. Best overall response (BOR) was defined as the best documented RECIST response from the index date until the earliest occurrence of radiographic progression, unequivocal clinical progression, initiation of a new systemic anticancer treatment, death, or the last evaluable tumor assessment. Tumor assessments performed after initiation of a new systemic anticancer treatment were not used to determine BOR. Patients without any evaluable post-baseline tumor assessment and without sufficient information to establish progressive disease were classified as not evaluable. Objective response rate (ORR) was defined as the proportion of patients with complete response or partial response, and disease control rate (DCR) as the proportion with complete response, partial response, or stable disease.

Follow-up information was obtained from outpatient records, inpatient records, imaging reports, treatment records, and documented follow-up information. Radiographic progression according to RECIST version 1.1 was used whenever evaluable imaging was available. In the absence of an evaluable radiographic assessment, clinical progression was recorded only when the treating oncologist explicitly documented unequivocal deterioration attributable to cervical cancer and the deterioration resulted in permanent discontinuation or change of the current anticancer regimen. Supporting evidence included newly developed or clearly worsening cancer-related symptoms or signs, new malignant bleeding, malignant pleural effusion or ascites, tumor-related urinary or gastrointestinal obstruction, deterioration in organ function attributable to tumor involvement, development of a clinically evident new tumor lesion, or sustained deterioration in performance status attributable to cancer. Deterioration attributable primarily to treatment toxicity, infection, or a non-malignant comorbidity was not classified as disease progression. For PFS, follow-up began on the date of the first administration of the index treatment; patients without progression or death were censored at the last valid tumor assessment, defined as the most recent post-baseline assessment with sufficient radiologic or clinical information to establish progression status before data cutoff or censoring. For overall survival, follow-up likewise began on the first administration date. Death status and the last confirmed survival date were ascertained from inpatient and outpatient records and documented follow-up information, and patients without documented death were censored at the last confirmed follow-up. Patients were considered lost to follow-up if no subsequent clinical, imaging, or contact record was available after treatment initiation and progression or survival status could not be confirmed.

### 2.5 Study Endpoints

The primary endpoint was progression-free survival, defined as the time from the date of the first administration of the index treatment to radiographic progression, documented clinical progression meeting the criteria specified above, or death from any cause, whichever occurred first. Secondary endpoints included overall survival, objective response rate, disease control rate, and treatment-related adverse events. Overall survival was defined as the time from the date of the first administration of the index treatment to death from any cause.

Treatment-related adverse events were graded according to Common Terminology Criteria for Adverse Events version 5.0. Safety endpoints included any treatment-related adverse event, grade 3–5 treatment-related adverse event, grade 3–5 myelosuppression, hypertension, proteinuria, thyroid dysfunction, dose modification or interruption, and treatment discontinuation. Early clinical benefit was assessed at the first post-baseline tumor evaluation after completion of two treatment cycles. Early clinical benefit was defined as complete response, partial response, or stable disease according to RECIST version 1.1 at that assessment; progressive disease was classified as absence of early clinical benefit. Patients without an evaluable tumor assessment after two treatment cycles were classified as not evaluable for early clinical benefit and were excluded from this exploratory comparison. Other post-baseline clinical markers included a reduction in squamous cell carcinoma antigen (SCC-Ag) of at least 50% from baseline, maintenance therapy, and treatment-emergent toxicity phenotypes. These variables were analyzed exclusively as exploratory post-treatment markers and were not entered into the primary treatment-effect model because their ascertainment occurred after initiation of the index treatment.

### 2.6 Statistical Analysis

Continuous variables were summarized as mean with standard deviation or median with interquartile range according to distributional characteristics, and categorical variables were summarized as number and percentage. Between-group comparisons were performed using Student’s *t* test, Mann-Whitney U test, chi-square test, or Fisher’s exact test, as appropriate. Standardized mean differences (SMDs) were used to assess baseline imbalance.

Progression-free survival and overall survival were analyzed using the Kaplan-Meier method, and differences between groups were compared using the log-rank test. Cox proportional hazards models were used to estimate hazard ratios and 95% confidence intervals. The primary progression-free survival model was a full-cohort baseline-adjusted Cox model including treatment group, liver metastasis, log1p-transformed baseline NLR, and recurrence field. A residual imbalance-adjusted sensitivity model additionally included Hb and prior PD-1/PD-L1 therapy. Proportional hazards assumptions were evaluated using Schoenfeld residual tests.

Propensity scores were estimated using logistic regression with treatment group as the dependent variable. The reduced propensity score model included age, ECOG PS, current treatment line, initial FIGO stage, and disease status. Propensity score methods included 1:1 nearest-neighbor propensity score matching (PSM), trimmed inverse probability of treatment weighting (IPTW), and overlap weighting. Cox analysis in the matched cohort used robust standard errors clustered by matched pair, and weighted Cox analyses used robust standard errors. Effective sample size (ESS) was reported for weighted analyses. Covariate balance after propensity score adjustment was evaluated using absolute SMDs.

Binary tumor response and safety outcomes were analyzed using odds ratios with 95% confidence intervals. Fisher’s exact test was used for low-frequency binary safety endpoints. Baseline subgroup consistency analyses for progression-free survival were performed using pretreatment baseline strata, including age, ECOG PS, current treatment line, liver metastasis, prior PD-1/PD-L1 therapy, and baseline NLR. Treatment-by-subgroup interaction terms were used to evaluate heterogeneity across subgroups.

Patients with missing key baseline or outcome information were excluded during cohort construction. Unknown or not-tested categories, including tumor grade and PD-L1 CPS, were retained as explicit descriptive categories. No multiple imputation was performed. Extreme or implausible values were checked against source records and corrected only when source-record errors were confirmed. Model influence, collinearity, propensity score range, common support, weight distribution, effective sample size, covariate balance, zero-cell status, and finite logistic coefficients were examined for the finalized models. No formal adjustment for multiple testing was applied to secondary, safety, subgroup, or exploratory analyses; corresponding *p* values were considered nominal and were interpreted as supportive or hypothesis-generating rather than confirmatory. All analyses were performed using R software, version 4.5.2.

## 3 Results

### 3.1 Cohort Construction and Analysis Populations

The cohort flow in Fig. 1 shows that the retrospective cohort was constructed from screened patients after eligibility assessment. Eligible patients were allocated to camrelizumab plus famitinib or control according to the actual treatment received, and the primary analysis set, safety analysis set, and propensity score-based analysis sets were defined before outcome analysis.

**Figure 1:**
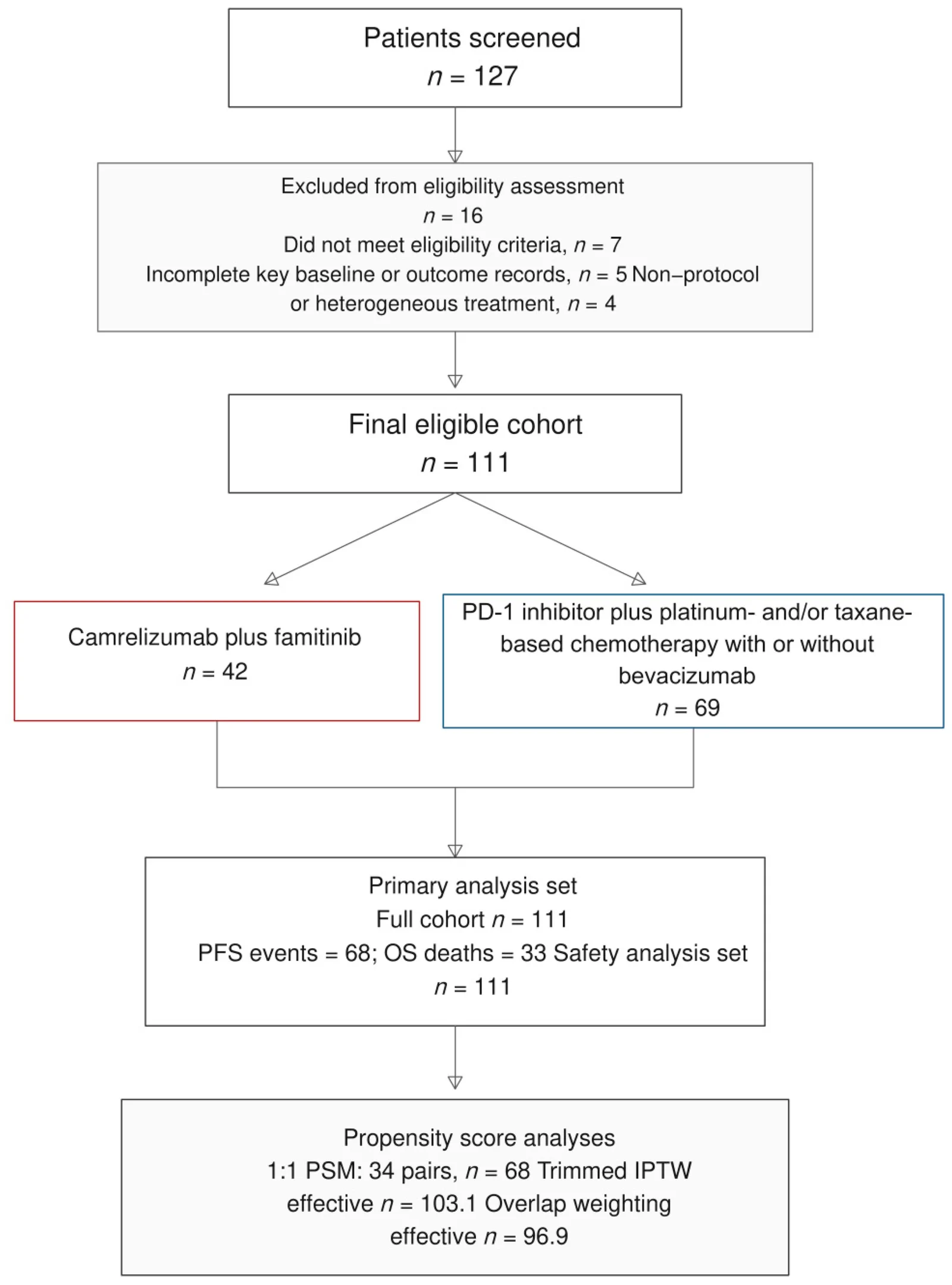
**Study design and cohort construction**. This flow diagram shows the construction of the single-center retrospective cohort, treatment-group allocation, major exclusion categories, primary analysis set, safety analysis set, and propensity score-based analysis sets.

### 3.2 Baseline Characteristics and Propensity Score Balance

Table 1 summarizes the baseline characteristics of the full cohort. Prior PD-1/PD-L1 therapy was more frequent in the camrelizumab plus famitinib group (31.0% vs. 13.0%; *p* = 0.022), and Hb was higher (116.4 ± 10.6 vs. 110.8 ± 11.6 g/L; *p* = 0.011). Current treatment-line distribution also differed between groups (*p* = 0.037): first-line treatment was less frequent (42.9% vs. 63.8%) and third-line or later treatment was more frequent (21.4% vs. 7.2%) in the camrelizumab plus famitinib group. Comparisons of the remaining demographic, performance status, tumor baseline, metastatic-site, prior-treatment, and baseline laboratory or inflammatory-nutritional variables were not statistically significant (all *p* > 0.05).

**Table 1:** Baseline characteristics of the full cohort.

Characteristic	Level	Overall (*n* = 111)	Camrelizumab plus Famitinib (*n* = 42)	Control (*n* = 69)	*t*/*Z*/*χ*^2^/Fisher	*p* Value	Standardized Mean Difference (SMD)
Demographics and performance status							
Age, years	Mean ± SD	53.1 ± 8.7	54.3 ± 8.8	52.3 ± 8.6	−1.167	0.246	0.231
BMI, kg/m^2^	Mean ± SD	22.5 ± 2.6	22.3 ± 2.7	22.5 ± 2.6	0.433	0.666	0.086
ECOG PS, n (%)	0	28 (25.2)	10 (23.8)	18 (26.1)	0.095	0.954	0.053
	1	63 (56.8)	24 (57.1)	39 (56.5)			
	2	20 (18.0)	8 (19.0)	12 (17.4)			
Menopausal status, n (%)	Premenopausal	42 (37.8)	15 (35.7)	27 (39.1)	0.130	0.719	0.071
	Postmenopausal	69 (62.2)	27 (64.3)	42 (60.9)			
**Tumor baseline characteristics**							
Histology, n (%)	Squamous cell carcinoma	89 (80.2)	34 (81.0)	55 (79.7)	Fisher	>0.999	0.046
	Adenocarcinoma	13 (11.7)	5 (11.9)	8 (11.6)			
	Adenosquamous carcinoma	6 (5.4)	2 (4.8)	4 (5.8)			
	Other	3 (2.7)	1 (2.4)	2 (2.9)			
Tumor grade, n (%)	G1	11 (9.9)	4 (9.5)	7 (10.1)	Fisher	>0.999	0.025
	G2	52 (46.8)	20 (47.6)	32 (46.4)			
	G3	37 (33.3)	14 (33.3)	23 (33.3)			
	Unknown	11 (9.9)	4 (9.5)	7 (10.1)			
Initial FIGO stage, n (%)	I	14 (12.6)	5 (11.9)	9 (13.0)	0.159	0.984	0.057
	II	30 (27.0)	12 (28.6)	18 (26.1)			
	III	47 (42.3)	18 (42.9)	29 (42.0)			
	IV	20 (18.0)	7 (16.7)	13 (18.8)			
Disease status, n (%)	Persistent disease	22 (19.8)	8 (19.0)	14 (20.3)	0.037	0.982	0.031
	Recurrent disease	66 (59.5)	25 (59.5)	41 (59.4)			
	*De novo* metastatic disease	23 (20.7)	9 (21.4)	14 (20.3)			
Recurrence interval, months	Median [IQR]	14.3 [6.9, 24.8]	12.9 [6.3, 18.7]	15.6 [7.5, 25.3]	−0.882	0.377	0.199
Prior pelvic radiotherapy, n (%)	No	41 (36.9)	15 (35.7)	26 (37.7)	0.043	0.835	0.041
	Yes	70 (63.1)	27 (64.3)	43 (62.3)			
Recurrence field, n (%)	No prior radiotherapy	41 (36.9)	15 (35.7)	26 (37.7)	0.096	0.992	0.056
	In-field	21 (18.9)	8 (19.0)	13 (18.8)			
	Out-of-field	30 (27.0)	12 (28.6)	18 (26.1)			
	Mixed	19 (17.1)	7 (16.7)	12 (17.4)			
Distant metastasis, n (%)	No	39 (35.1)	13 (31.0)	26 (37.7)	0.519	0.471	0.142
	Yes	72 (64.9)	29 (69.0)	43 (62.3)			
Metastatic site count, n (%)	0	39 (35.1)	13 (31.0)	26 (37.7)	Fisher	0.827	0.142
	1	35 (31.5)	13 (31.0)	22 (31.9)			
	2	26 (23.4)	11 (26.2)	15 (21.7)			
	≥3	11 (9.9)	5 (11.9)	6 (8.7)			
Measurable disease, n (%)	Evaluable only	13 (11.7)	5 (11.9)	8 (11.6)	Fisher	>0.999	0.010
	Measurable	98 (88.3)	37 (88.1)	61 (88.4)			
**Key metastatic sites**							
Lung metastasis, n (%)	No	71 (64.0)	26 (61.9)	45 (65.2)	0.124	0.724	0.069
	Yes	40 (36.0)	16 (38.1)	24 (34.8)			
Liver metastasis, n (%)	No	91 (82.0)	35 (83.3)	56 (81.2)	0.084	0.773	0.057
	Yes	20 (18.0)	7 (16.7)	13 (18.8)			
Bone metastasis, n (%)	No	96 (86.5)	36 (85.7)	60 (87.0)	0.034	0.853	0.036
	Yes	15 (13.5)	6 (14.3)	9 (13.0)			
Lymph-node-only disease, n (%)	No	90 (81.1)	34 (81.0)	56 (81.2)	0.001	0.978	0.005
	Yes	21 (18.9)	8 (19.0)	13 (18.8)			
**Prior treatment and treatment line**							
Prior cervical cancer surgery, n (%)	No	52 (46.8)	19 (45.2)	33 (47.8)	0.070	0.791	0.052
	Yes	59 (53.2)	23 (54.8)	36 (52.2)			
Prior platinum therapy, n (%)	No	28 (25.2)	9 (21.4)	19 (27.5)	0.516	0.472	0.142
	Yes	83 (74.8)	33 (78.6)	50 (72.5)			
Prior bevacizumab, n (%)	No	94 (84.7)	34 (81.0)	60 (87.0)	0.726	0.394	0.164
	Yes	17 (15.3)	8 (19.0)	9 (13.0)			
Prior PD-1/PD-L1 therapy, n (%)	No	89 (80.2)	29 (69.0)	60 (87.0)	5.269	0.022	0.443
	Yes	22 (19.8)	13 (31.0)	9 (13.0)			
Current treatment line, n (%)	First-line	62 (55.9)	18 (42.9)	44 (63.8)	6.582	0.037	0.429
	Second-line	35 (31.5)	15 (35.7)	20 (29.0)			
	Third-line or later	14 (12.6)	9 (21.4)	5 (7.2)			
**Baseline laboratory and inflammatory-nutritional markers**							
SCC-Ag, ng/mL	Median [IQR]	24.38 [15.43, 43.70]	23.45 [12.59, 42.26]	25.94 [17.93, 44.12]	−1.161	0.247	0.184
Hb, g/L	Mean ± SD	112.9 ± 11.5	116.4 ± 10.6	110.8 ± 11.6	−2.600	0.011	0.508
Alb, g/L	Mean ± SD	38.1 ± 4.0	38.3 ± 3.8	38.0 ± 4.2	−0.381	0.704	0.074
LDH, U/L	Median [IQR]	222.0 [176.5, 290.8]	221.0 [183.0, 284.0]	223.0 [173.0, 307.5]	−0.035	0.975	0.136
NLR	Median [IQR]	4.06 [2.96, 5.19]	3.63 [2.96, 4.96]	4.36 [2.93, 5.51]	−1.107	0.270	0.224
PLR	Mean ± SD	230.1 ± 72.0	213.2 ± 72.3	240.3 ± 70.3	1.915	0.059	0.384
PNI	Mean ± SD	38.5 ± 4.5	39.0 ± 3.9	38.2 ± 4.8	−0.916	0.362	0.177
PD-L1 CPS, n (%)	<1	19 (17.1)	7 (16.7)	12 (17.4)	0.124	0.989	0.065
	1–9	29 (26.1)	11 (26.2)	18 (26.1)			
	≥10	22 (19.8)	9 (21.4)	13 (18.8)			
	Not tested	41 (36.9)	15 (35.7)	26 (37.7)			

Note: Data are shown as mean ± standard deviation (SD), median [interquartile range (IQR)], or n (%). Normality was assessed using the Shapiro-Wilk test. Between-group comparisons used Student’s *t* test, Mann-Whitney U test, chi-square test, or Fisher’s exact test as appropriate. The statistic column reports t, Z, χ^2^, or Fisher. Standardized mean difference (SMD) was used to quantify baseline imbalance.

Fig. 2A shows overlap in the reduced propensity score distributions between camrelizumab plus famitinib and control. Fig. 2B shows covariate balance before adjustment and after 1:1 propensity score matching, while Fig. 2C shows balance before adjustment and after trimmed IPTW and overlap weighting. Absolute standardized mean differences decreased after each propensity score procedure, with the weighted approaches showing smaller residual imbalance than matching.

Full propensity score diagnostics are provided in Table S1. Reduced propensity score covariate balance improved after trimmed IPTW and overlap weighting, whereas 1:1 propensity score matching retained residual imbalance for some reduced propensity score covariates. Among additional clinically relevant baseline covariates, residual imbalance remained after propensity score procedures, including prior PD-1/PD-L1 therapy, Hb, and platelet-to-lymphocyte ratio (PLR).

**Figure 2:**
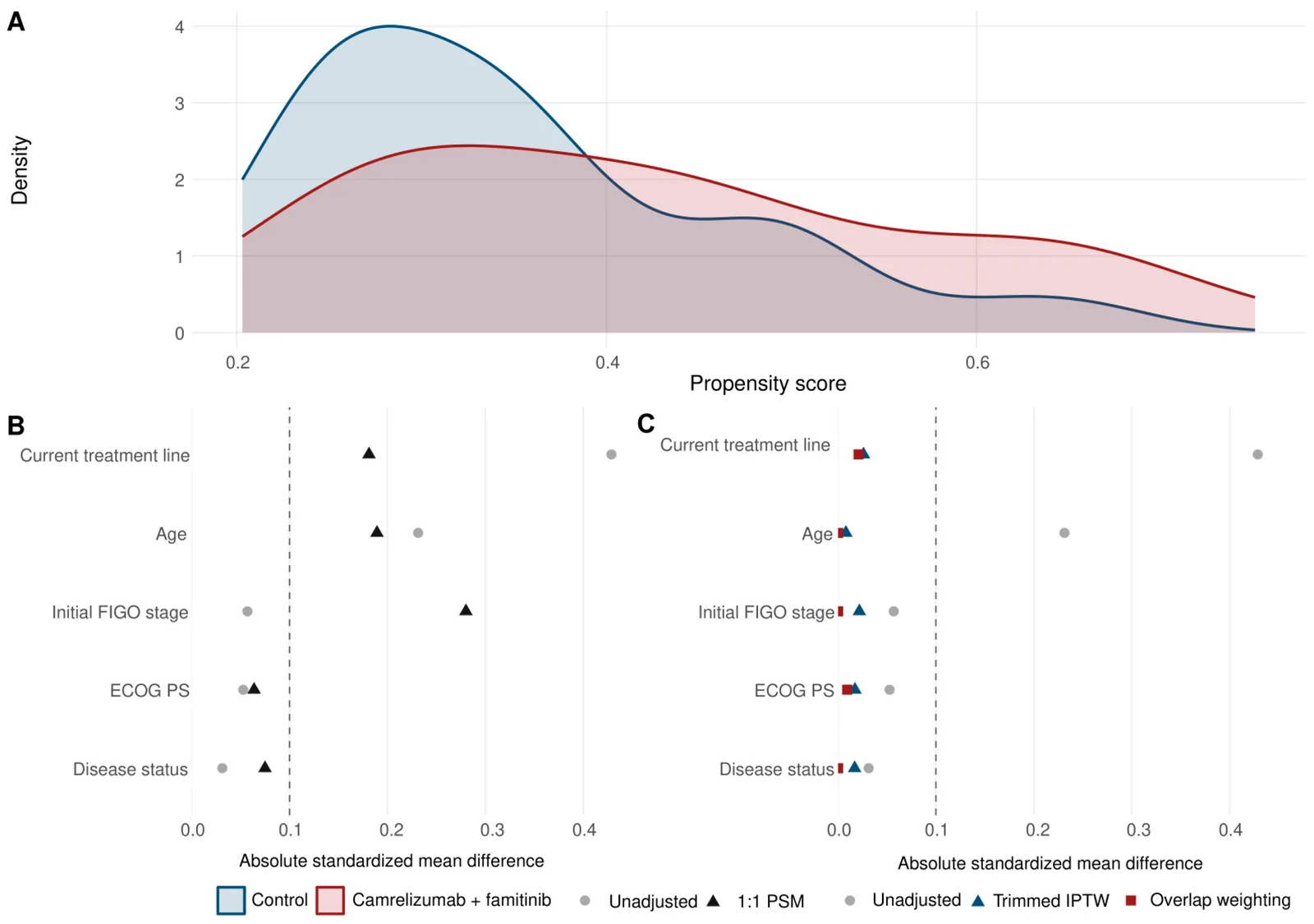
**Propensity score overlap and covariate balance**. (**A**) Propensity score distributions in the reduced propensity score model for the camrelizumab plus famitinib group and the control group. (**B**) Absolute standardized mean differences for reduced propensity score covariates before adjustment and after 1:1 propensity score matching. (**C**) Absolute standardized mean differences for reduced propensity score covariates before adjustment, after trimmed inverse probability of treatment weighting, and after overlap weighting. The vertical reference line indicates SMD = 0.10. Full balance diagnostics for clinically relevant baseline covariates are reported in Table S1.

### 3.3 Survival Outcomes

The Kaplan-Meier curves show longer PFS with camrelizumab plus famitinib in the full cohort (Fig. 3A; *p* = 0.026) and in the propensity score-matched cohort (Fig. 3B; *p* = 0.004). Overall survival did not differ significantly in either the full cohort (Fig. 3C) or the matched cohort (Fig. 3D; both *p* > 0.05).

**Figure 3:**
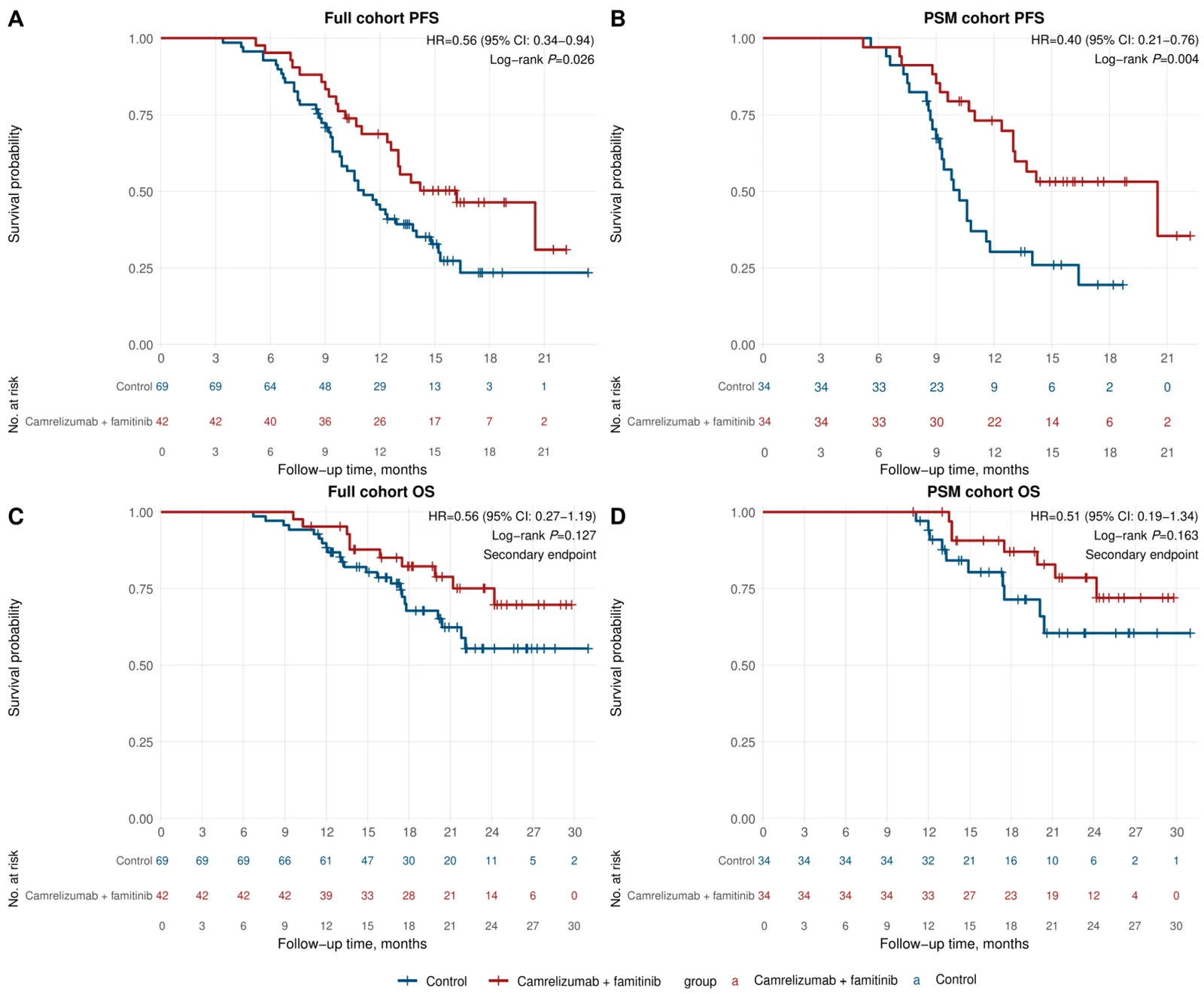
**Survival outcomes**. (**A**) Progression-free survival in the full cohort. (**B**) Progression-free survival in the propensity score-matched cohort. (**C**) Overall survival in the full cohort. (**D**) Overall survival in the propensity score-matched cohort. Kaplan-Meier curves include censoring marks and risk tables. PFS was the primary endpoint, and OS was analyzed as a secondary endpoint.

### 3.4 Primary and Robustness Analyses for Progression-Free Survival

The primary and robustness analyses for PFS are summarized in Table 2. PFS favored camrelizumab plus famitinib in the unadjusted Cox model (*p* = 0.028), residual imbalance-adjusted Cox model (*p* = 0.041), 1:1 PSM Cox model (*p* = 0.010), trimmed IPTW Cox model (*p* = 0.006), and overlap-weighted Cox model (*p* = 0.005). The baseline-adjusted Cox model comparison was not statistically significant (*p* > 0.05).

**Table 2:** Primary and robustness analyses for Progression-Free survival.

Analysis Strategy	Analysis Set	n or ESS	Events	Hazard Ratio (HR)	95% Confidence Interval (CI)	*p* Value
Unadjusted Cox	Full cohort	111	68	0.56	0.34–0.94	0.028
Baseline-adjusted Cox	Full cohort	111	68	0.60	0.36–1.03	0.062
Residual imbalance-adjusted Cox	Full cohort	111	68	0.56	0.32–0.98	0.041
1:1 PSM Cox	Matched cohort	68	40	0.40	0.20–0.80	0.010
Trimmed IPTW Cox	Weighted full cohort	103.1	68	0.49	0.30–0.82	0.006
Overlap-weighted Cox	Weighted full cohort	96.9	68	0.48	0.29–0.80	0.005

Note: Hazard ratios (HRs) compare camrelizumab plus famitinib with the control regimen; 95% confidence intervals (CIs) are reported for all effect estimates. The full-cohort baseline-adjusted Cox model was used as the primary inferential model. The residual imbalance-adjusted model additionally included Hb and prior PD-1/PD-L1 exposure. PSM, trimmed IPTW, and overlap weighting were used as robustness analyses. Effective sample size is reported for weighted analyses.

Consistent with Table 2, Fig. 4A shows that PFS treatment-effect estimates favored camrelizumab plus famitinib in the unadjusted Cox (*p* = 0.028), residual imbalance-adjusted Cox (*p* = 0.041), 1:1 PSM Cox (*p* = 0.010), trimmed IPTW Cox (*p* = 0.006), and overlap-weighted Cox analyses (*p* = 0.005), whereas the baseline-adjusted Cox estimate was not statistically significant (*p* = 0.062). Fig. 4B shows the baseline subgroup analyses; all treatment-by-subgroup interaction tests were non-significant (all *p* > 0.05).

**Figure 4:**
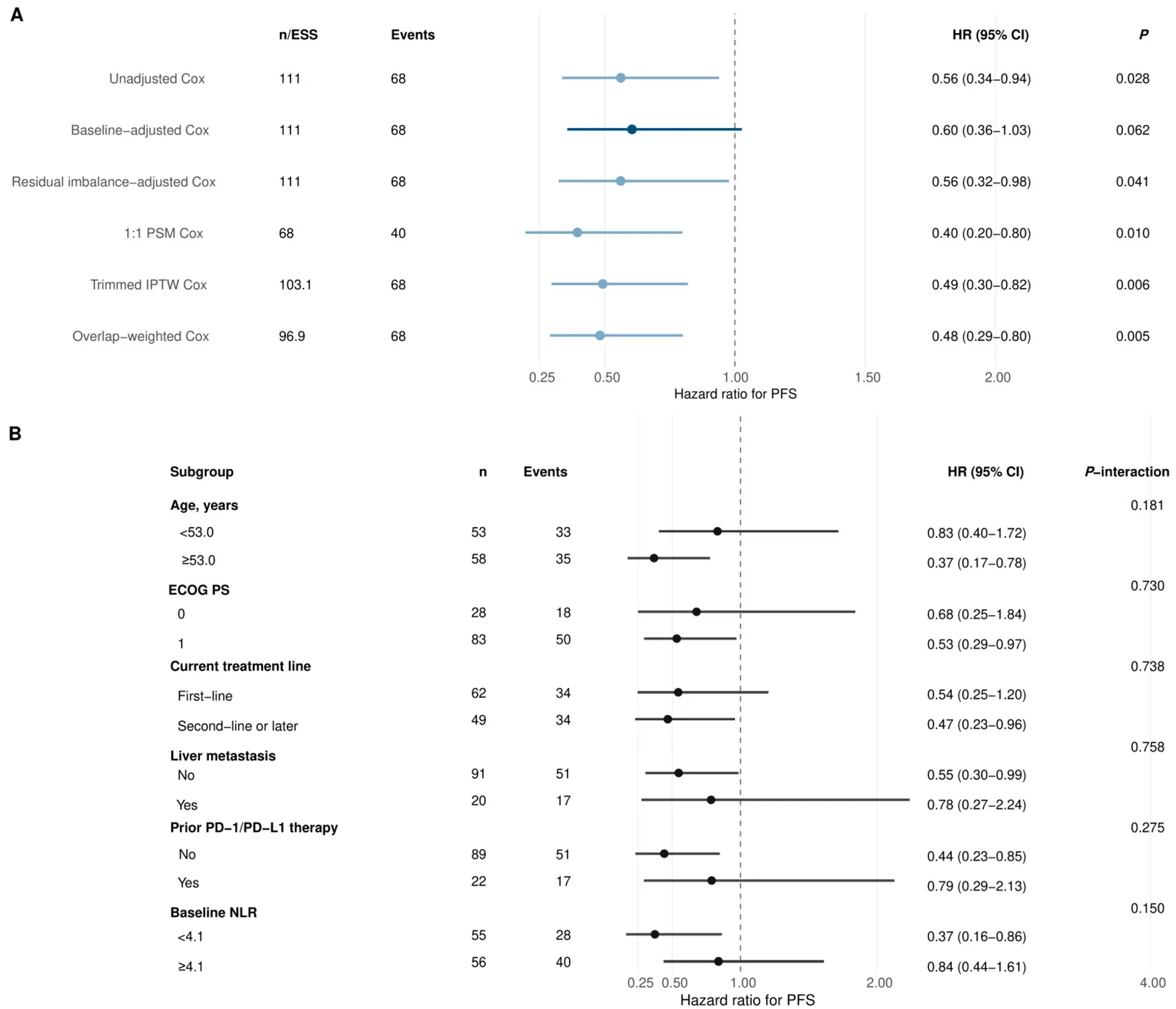
**Robustness and baseline subgroup consistency of Progression-Free survival**. (**A**) Forest plot of progression-free survival treatment-effect estimates across unadjusted Cox, baseline-adjusted Cox, residual imbalance-adjusted Cox, 1:1 propensity score-matched Cox, trimmed IPTW Cox, and overlap-weighted Cox analyses. (**B**) Baseline subgroup analysis for progression-free survival using treatment-prebaseline clinical strata. The subgroup panel reports treatment-effect estimates and interaction *p* values.

### 3.5 Baseline-Adjusted Cox Model for Progression-Free Survival

In the baseline-adjusted Cox model for PFS presented in Table 3, liver metastasis (*p* = 0.001) and baseline NLR (*p* = 0.037) were associated with shorter PFS. Treatment group and recurrence field comparisons were not statistically significant (all *p* > 0.05).

**Table 3:** Baseline-Adjusted Cox model for Progression-Free survival.

Variable	Comparison or Unit	Hazard Ratio (HR)	95% Confidence Interval (CI)	Wald Chi-Square	*p* Value
Treatment group	Camrelizumab plus famitinib vs. control	0.60	0.36–1.03	3.47	0.062
Liver metastasis	Yes vs. no	2.62	1.47–4.66	10.75	0.001
Baseline NLR	Per 1-unit increase in log1p-transformed NLR	2.42	1.05–5.56	4.35	0.037
Recurrence field	Overall Wald test			6.72	0.081
Recurrence field	In-field vs. no prior radiotherapy	1.64	0.88–3.07	2.39	0.122
Recurrence field	Out-of-field vs. no prior radiotherapy	0.63	0.32–1.25	1.76	0.185
Recurrence field	Mixed vs. no prior radiotherapy	1.31	0.62–2.76	0.49	0.484

Note: The model included baseline covariates only. Hazard ratios (HRs) and 95% confidence intervals (CIs) are reported; the treatment HR compares camrelizumab plus famitinib with the control regimen. Baseline NLR was log1p-transformed before modeling. Recurrence field was modeled as a categorical baseline variable with no prior radiotherapy as the reference category. Proportional hazards and influence diagnostics are reported separately in the supplementary diagnostics.

### 3.6 Treatment Exposure and Tumor Response Outcomes

Treatment exposure and tumor response outcomes are shown in Table 4. Current treatment-line distribution differed between groups (*p* = 0.037), with first-line treatment less frequent (42.9% vs. 63.8%) and third-line or later treatment more frequent (21.4% vs. 7.2%) in the camrelizumab plus famitinib group. Exposure to platinum agents, taxanes, and bevacizumab during the index treatment period differed significantly between groups (all *p* < 0.001). DCR was higher with camrelizumab plus famitinib (85.7% vs. 68.1%; *p* = 0.038). Local therapy during treatment, best overall response, and ORR did not differ significantly.

**Table 4:** Treatment exposure and tumor response outcomes.

Outcome	Category	Camrelizumab plus Famitinib	Control	Statistic or Effect	95% Confidence Interval (CI)	*p* Value
Treatment exposure						
Current treatment line	First-line	18 (42.9)	44 (63.8)	*χ*^2^ = 6.582		0.037
	Second-line	15 (35.7)	20 (29.0)			
	Third-line or later	9 (21.4)	5 (7.2)			
Platinum agent	None	38 (90.5)	5 (7.2)	Fisher		<0.001
	Cisplatin	2 (4.8)	31 (44.9)			
	Carboplatin	1 (2.4)	31 (44.9)			
	Nedaplatin	1 (2.4)	2 (2.9)			
Taxane agent	None	36 (85.7)	4 (5.8)	Fisher		<0.001
	Paclitaxel	3 (7.1)	30 (43.5)			
	Nab-paclitaxel	2 (4.8)	27 (39.1)			
	Docetaxel	1 (2.4)	8 (11.6)			
Bevacizumab use	No	41 (97.6)	41 (59.4)	*χ*^2^ = 19.738		<0.001
	Yes	1 (2.4)	28 (40.6)			
Local therapy during treatment	No	33 (78.6)	58 (84.1)	*χ*^2^ = 0.532		0.466
	Yes	9 (21.4)	11 (15.9)			
**Tumor response**						
Best overall response	CR	2 (4.8)	2 (2.9)	Fisher		0.137
	PR	20 (47.6)	22 (31.9)			
	SD	14 (33.3)	23 (33.3)			
	PD	5 (11.9)	21 (30.4)			
	Not evaluable	1 (2.4)	1 (1.4)			
ORR	Yes	22 (52.4)	24 (34.8)	OR = 2.06	0.94–4.51	0.068
DCR	Yes	36 (85.7)	47 (68.1)	OR = 2.81	1.03–7.65	0.038

Note: Data are shown as n (%). Between-group comparisons used chi-square tests or Fisher’s exact tests according to expected cell counts. Odds ratios (ORs) compare camrelizumab plus famitinib with the control regimen for objective response rate (ORR) and disease control rate (DCR); 95% confidence intervals (CIs) are reported for the ORs. Tumor response outcomes were derived according to RECIST-based best response categories. This table summarizes treatment exposure and short-term tumor response.

### 3.7 Treatment-Related Adverse Events

The treatment-related adverse event profile is summarized in Table 5. Grade 3–5 myelosuppression (*p* = 0.010) was lower in the camrelizumab plus famitinib group than in the control group, whereas hypertension (*p* = 0.023) was higher in the camrelizumab plus famitinib group. Any TRAE, grade 3–5 TRAE, dose modification or interruption, treatment discontinuation, proteinuria, and thyroid dysfunction comparisons were not statistically significant (all *p* > 0.05).

**Table 5:** Treatment-Related adverse events.

Adverse Event or Safety Outcome	Camrelizumab plus Famitinib	Control	Odds Ratio (OR)	95% Confidence Interval (CI)	*p* Value
Overall safety					
Any TRAE	34 (81.0)	59 (85.5)	0.72	0.23–2.33	0.599
Grade 3–5 TRAE	8 (19.0)	26 (37.7)	0.39	0.14–1.04	0.055
Dose modification or interruption	14 (33.3)	18 (26.1)	1.41	0.56–3.53	0.517
Treatment discontinuation	22 (52.4)	46 (66.7)	0.55	0.23–1.30	0.162
**Myelosuppression-related events**					
Grade 3–5 myelosuppression	4 (9.5)	22 (31.9)	0.23	0.05–0.75	0.010
**Antiangiogenic or immune-related events**					
Hypertension	16 (38.1)	12 (17.4)	2.89	1.11–7.78	0.023
Proteinuria	11 (26.2)	8 (11.6)	2.68	0.88–8.54	0.068
Thyroid dysfunction	8 (19.0)	10 (14.5)	1.38	0.43–4.33	0.599

Note: Data are shown as n (%). Exact odds ratios (ORs), exact 95% confidence intervals (CIs), and *p* values were calculated using Fisher’s exact test for binary safety endpoints. ORs compare camrelizumab plus famitinib with the control regimen. Treatment-related adverse events were recorded according to prespecified toxicity grading criteria.

### 3.8 Benefit-Risk Profile and Exploratory Post-Baseline Clinical Markers

Fig. 5A summarizes the key response and safety effect estimates: DCR was higher (*p* = 0.038), grade 3–5 myelosuppression was lower (*p* = 0.010), and hypertension was higher (*p* = 0.023) with camrelizumab plus famitinib. In the exploratory post-baseline clinical marker analysis (Fig. 5B), SCC-Ag decrease ≥50% and maintenance therapy were associated with longer subsequent PFS (both *p* < 0.001), whereas early clinical benefit was not. In the post-baseline toxicity analysis (Fig. 5C), hypertension, proteinuria, thyroid dysfunction, and dose modification/interruption were not significantly associated with subsequent PFS (all *p* > 0.05).

**Figure 5:**
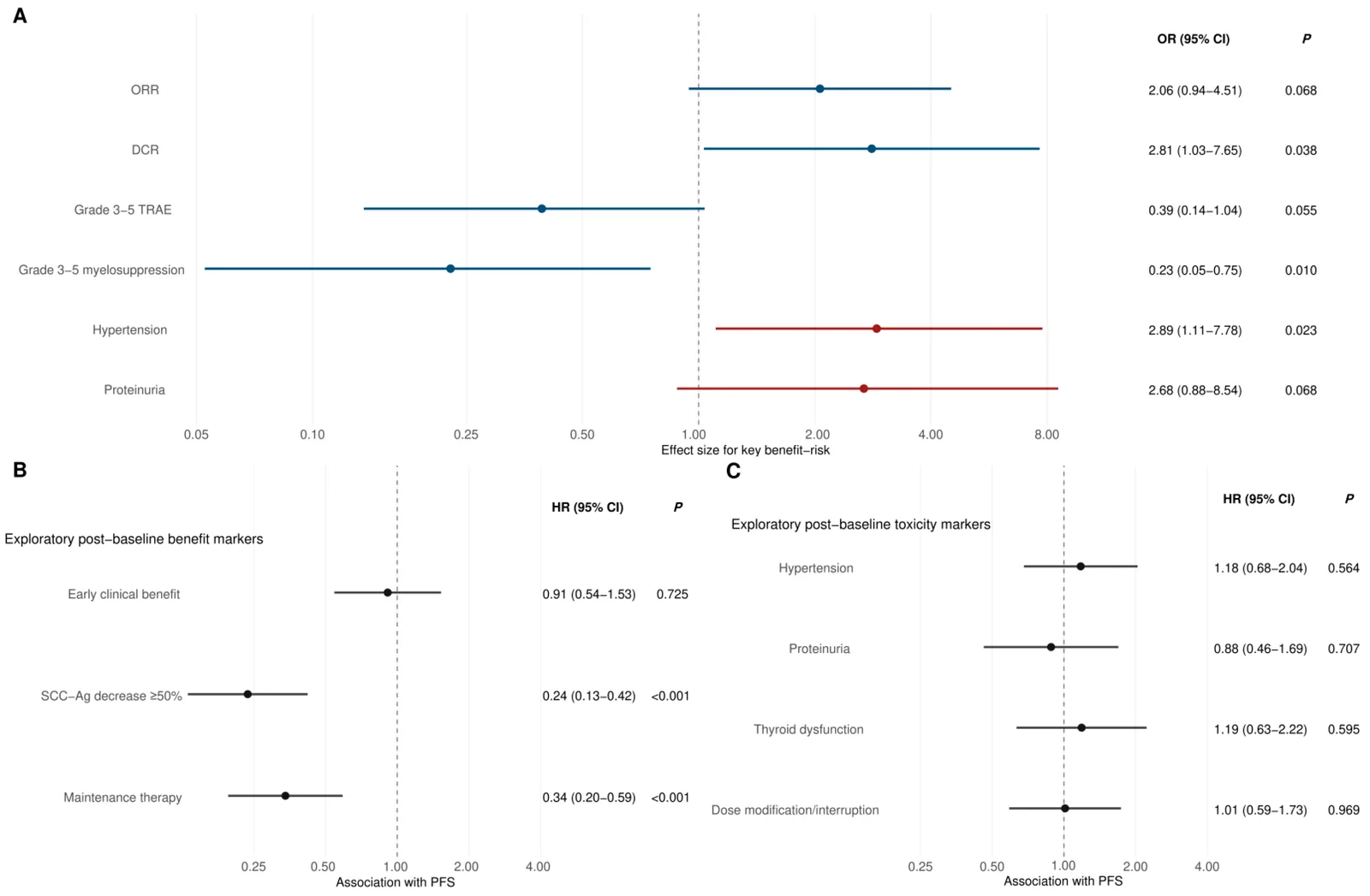
**Benefit-Risk profile and exploratory Post-Baseline clinical markers**. (**A**) Effect-size overview for key tumor response and safety endpoints, including ORR, DCR, grade 3–5 TRAEs, grade 3–5 myelosuppression, hypertension, and proteinuria. (**B**) Associations between post-baseline clinical benefit markers and subsequent PFS. (**C**) Associations between post-baseline toxicity phenotypes and subsequent PFS.

### 3.9 Model Assumptions and Numerical Stability Diagnostics

Model assumptions and numerical stability diagnostics are reported in Table S2. Proportional hazards tests for the PFS baseline-adjusted Cox model, treatment effect, liver metastasis, baseline NLR, recurrence field, and the secondary OS Cox model were not statistically significant (all *p* > 0.05). Influence, collinearity, propensity score, weighting, and binary outcome numerical stability diagnostics were classified as pass or acceptable, except reduced propensity score covariate balance after PSM, which was classified as caution.

## 4 Discussion

### 4.1 Principal Findings

In this single-center retrospective cohort study, camrelizumab plus famitinib was evaluated against PD-1 inhibitor plus platinum- and/or taxane-based chemotherapy with or without bevacizumab in patients with persistent, recurrent, or metastatic cervical cancer (CC). The main signal was observed in PFS: the unadjusted, residual imbalance-adjusted, PSM, trimmed IPTW, and overlap-weighted analyses consistently favored camrelizumab plus famitinib, whereas the prespecified baseline-adjusted Cox model showed a directionally favorable but statistically non-significant estimate. DCR was higher with camrelizumab plus famitinib, while ORR did not differ significantly. The safety profile also differed between groups, with less grade 3–5 myelosuppression but more hypertension in the camrelizumab plus famitinib group. OS did not show a significant difference, which is not unexpected in a cohort with limited event maturity and heterogeneous subsequent treatment exposure.

The current systemic treatment landscape for recurrent or metastatic CC has moved from chemotherapy alone toward chemotherapy-immunotherapy and antiangiogenic combinations. Platinum-taxane chemotherapy remains an important backbone, and the addition of bevacizumab has provided survival improvement in selected patients, but hematologic toxicity and cumulative treatment burden remain relevant in practice [16]. The incorporation of PD-1 or PD-L1 blockade into first-line systemic therapy has further improved outcomes in advanced CC, especially in biomarker-selected or immunologically responsive populations [17]. Against this background, an oral antiangiogenic TKI combined with PD-1 blockade offers a different treatment pattern from chemotherapy-based combinations, and the present study adds comparative data for this specific regimen without assuming equivalence to randomized evidence.

### 4.2 Interpretation of Efficacy and Disease Control

The PFS results should be read with caution but not dismissed. In the main baseline-adjusted Cox model, the treatment effect did not reach conventional statistical significance, while several robustness approaches yielded significant estimates in the same direction. This pattern suggests that the observed PFS advantage is sensitive to analytic strategy but directionally stable across multiple methods. Such a finding is plausible in a modest-sized retrospective cohort, particularly when the treated group had higher prior PD-1/PD-L1 exposure and later-line treatment distribution at baseline. In this context, the value of PSM, IPTW, and overlap weighting is not to prove causality, but to examine whether the treatment signal is preserved when treatment selection is handled differently [18].

The higher DCR, rather than a statistically significant ORR difference, is also clinically coherent for an antiangiogenic-immunotherapy combination. Antiangiogenic agents may contribute to tumor growth control without necessarily producing rapid tumor shrinkage in all patients, and immune-based regimens can show delayed or heterogeneous response kinetics [19]. This helps explain why DCR and PFS may move in the same direction even when ORR does not reach statistical significance. Still, the absence of a significant ORR difference should temper the language: the data support better disease control, not a broad claim of superior radiographic response.

The lack of a significant OS difference requires a similar level of restraint. In recurrent or metastatic CC, OS can be affected by subsequent therapies, treatment discontinuation, later-line access, and follow-up duration. Randomized phase III evidence has demonstrated an OS benefit from adding pembrolizumab to platinum-based chemotherapy with or without bevacizumab in persistent, recurrent, or metastatic cervical cancer, with analyses prespecified across PD-L1-defined populations [20]. Therefore, in the present study, PFS and DCR are more informative than OS for evaluating the observed treatment signal, while OS should remain a secondary and immature endpoint.

### 4.3 Safety Profile and Treatment Tolerability

The safety findings are consistent with the expected biological difference between a TKI-based regimen and a chemotherapy-based regimen. The lower incidence of grade 3–5 myelosuppression in the camrelizumab plus famitinib group likely reflects reduced exposure to platinum-taxane chemotherapy, whereas the higher incidence of hypertension aligns with antiangiogenic pathway inhibition. VEGF pathway blockade is known to affect vascular tone, endothelial function, and renal microvascular regulation, which can lead to hypertension and proteinuria during treatment [21]. In this study, hypertension was increased, proteinuria was numerically higher but not statistically significant, and grade 3–5 TRAEs did not differ significantly. This pattern supports the interpretation of a toxicity shift rather than a simple “safer” or “more toxic” profile.

This distinction is important. Chemotherapy-based immunotherapy combinations tend to carry hematologic toxicity, while antiangiogenic TKIs are more often associated with hypertension, proteinuria, hand-foot reactions, mucosal effects, and dose modification in broader oncology settings [22]. The present dataset only captured selected safety endpoints, so it would be inappropriate to generalize beyond the recorded toxicity spectrum. What can be stated is narrower and more defensible: camrelizumab plus famitinib was associated with less severe myelosuppression and more hypertension than the control regimen in this cohort.

### 4.4 Baseline Risk Factors and Post-Baseline Clinical Markers

Liver metastasis and higher baseline NLR were associated with shorter PFS in the baseline-adjusted Cox model. Both variables have plausible clinical meaning. Liver metastasis often indicates greater systemic tumor dissemination and may be associated with reduced response to immunotherapy in several tumor types, partly because the liver can promote immune tolerance and alter systemic antitumor immunity [23]. NLR is a routinely available inflammatory marker, and elevated NLR has been linked to poorer outcomes in multiple cancers, including patients treated with immune checkpoint inhibitors (ICIs) [24]. In this study, these variables were not used to build a predictive model; rather, they helped adjust for baseline disease aggressiveness and inflammatory status when estimating the treatment association.

The exploratory post-baseline analyses showed that SCC-Ag decrease ≥50% and maintenance therapy were associated with longer subsequent PFS. These findings are clinically reasonable but should not be interpreted as baseline predictors or mechanistic proof. SCC-Ag can reflect tumor burden and treatment response in squamous cervical cancer, but post-treatment decline is partly a consequence of early disease control [25]. Maintenance therapy is also a post-baseline variable and may be influenced by treatment tolerance, absence of early progression, physician decision-making, and patient preference. For this reason, the present analysis appropriately kept these variables outside the primary Cox model. They are best viewed as descriptive clinical markers that may help characterize patients who remain on a favorable treatment course.

Treatment-emergent hypertension and proteinuria were not clearly associated with subsequent PFS in the exploratory analyses. In other antiangiogenic settings, on-treatment hypertension has sometimes been proposed as a pharmacodynamic marker, but findings are inconsistent and vulnerable to time-dependent and selection biases [26]. The current results do not support using these toxicity events as surrogate markers of benefit in this cohort. This is a useful negative signal, because it prevents overinterpretation of adverse events as evidence of efficacy.

### 4.5 Methodological Considerations

A strength of this study is the use of several complementary analytic strategies rather than relying on a single matched comparison. The full cohort preserved sample size and event information, the baseline-adjusted Cox model provided the main prespecified inference, and the residual imbalance-adjusted model addressed clinically relevant remaining imbalance. PSM, trimmed IPTW, and overlap weighting tested the stability of the treatment association under different assumptions about the treatment-selection process. Overlap weighting is particularly useful when treatment groups differ but still share a clinically meaningful common support region [27].

Another strength is the separation of baseline covariates from post-baseline markers. This is not just a statistical detail. In retrospective treatment studies, including variables such as early response, SCC-Ag decline, maintenance therapy, or toxicity events in a primary treatment-effect model can introduce time-dependent bias or post-treatment adjustment bias [28]. By keeping these markers exploratory, the analysis avoids overstating them as determinants of treatment efficacy. The model diagnostics also add reassurance that the finalized Cox and binary outcome analyses were not dominated by obvious proportional hazards violations, extreme weights, collinearity, or zero-cell instability.

### 4.6 Limitations

Several limitations should be acknowledged. First, the retrospective single-center design inevitably carries risks of selection bias, information bias, and residual confounding. Although propensity score methods and residual imbalance adjustment were applied, unmeasured factors such as physician preference, treatment accessibility, tumor molecular features, and subsequent therapies could not be fully controlled [29]. Second, the sample size was modest, especially in the camrelizumab plus famitinib group, which limits precision in subgroup analyses and may explain why some estimates were directionally consistent but not statistically significant. Third, PD-L1 CPS was not available for all patients, and the study could not fully evaluate biomarker-defined immunotherapy benefit. Fourth, OS follow-up was relatively immature, so OS should not be used as the main basis for judging comparative efficacy.

The safety analysis was also limited by the endpoints captured in the finalized dataset. Selected TRAEs were analyzed consistently, but less frequent or more granular toxicities were not evaluated in detail. In addition, dose intensity, duration of famitinib exposure, and reasons for dose modification were not modeled as time-varying factors. Finally, the exploratory marker analyses were hypothesis-generating. SCC-Ag decline, maintenance therapy, and toxicity phenotypes occur after treatment initiation and therefore cannot be interpreted in the same way as baseline prognostic variables [30]. Because no formal multiplicity adjustment was applied to secondary, safety, subgroup, or exploratory analyses, their *p* values should be regarded as nominal and interpreted as supportive or hypothesis-generating.

Overall, the study provides a cautious but clinically relevant comparison of camrelizumab plus famitinib with a commonly used PD-1 inhibitor plus platinum- and/or taxane-based chemotherapy with or without bevacizumab in persistent, recurrent, or metastatic CC. The findings support further evaluation of this chemotherapy-sparing immunotherapy-antiangiogenic strategy, particularly with larger cohorts, longer OS follow-up, and more complete biomarker information.

## 5 Conclusions

Camrelizumab plus famitinib showed a favorable progression-free survival direction and higher disease control compared with PD-1 inhibitor plus platinum- and/or taxane-based chemotherapy with or without bevacizumab in patients with persistent, recurrent, or metastatic CC. The regimen was associated with lower grade 3–5 myelosuppression and higher hypertension, indicating a distinct safety profile rather than a uniform reduction in treatment-related toxicity. Baseline liver metastasis and higher NLR were associated with shorter PFS, while post-baseline SCC-Ag decrease and maintenance therapy were linked to longer subsequent PFS in exploratory analyses. These findings support the hypothesis that camrelizumab plus famitinib has clinically relevant activity and a distinguishable toxicity pattern in this patient population.

## Data Availability

Restrictions apply to the availability of these data due to patient privacy and ethical requirements. De-identified data may be available from the corresponding author upon reasonable request and with approval from the relevant institutional authority.

## Supplementary Materials

The supplementary material is available online at https://www.techscience.com/doi/10.32604/or.2026.088774/s1. Table S1: Full Propensity Score Diagnostics. Table S2: Model Assumptions and Numerical Stability Diagnostics.

## Abbreviations

Alb, albumin; BMI, body mass index; CC, cervical cancer; CI, confidence interval; CPS, combined positive score; CR, complete response; CTCAE, common terminology criteria for adverse events; DCR, disease control rate; ECOG PS, eastern cooperative oncology group performance status; ESS, effective sample size; FIGO, international federation of gynecology and obstetrics; Hb, hemoglobin; HR, hazard ratio; ICI, immune checkpoint inhibitor; IPTW, inverse probability of treatment weighting; IQR, interquartile range; LDH, lactate dehydrogenase; NLR, neutrophil-to-lymphocyte ratio; OR, odds ratio; ORR, objective response rate; OS, overall survival; PD, progressive disease; PD-1, programmed cell death protein 1; PD-L1, programmed death ligand 1; PFS, progression-free survival; PLR, platelet-to-lymphocyte ratio; PNI, prognostic nutritional index; PR, partial response; PSM, propensity score matching; RECIST, response evaluation criteria in solid tumors; SCC-Ag, squamous cell carcinoma antigen; SD, stable disease or standard deviation; SMD, standardized mean difference; TKI, tyrosine kinase inhibitor; TRAE, treatment-related adverse event; VEGF, vascular endothelial growth factor; VEGFR, vascular endothelial growth factor receptor.
