# Supplementary material for: Clinical Outcomes and Safety Profile of Camrelizumab Plus Famitinib in Persistent, Recurrent, or Metastatic Cervical Cancer
Source: Oncol Res. 2026 Oct 28;34(11):21. doi: 10.32604/or.2026.088774 (PMC13732342; doi:10.32604/or.2026.088774)
Supplement: Supplementary file 1 [file OncolRes-34-88774-s001.zip › TSP_OR_88774_Supplementary_Material.docx]

**Supplementary Tables**

**Table S1. Full Propensity Score Diagnostics**

| **Covariate** | Unadjusted standardized mean difference (SMD) | **After 1:1 PSM SMD** | **Trimmed IPTW SMD** | **Overlap weighting SMD** |
| --- | --- | --- | --- | --- |
| **Reduced propensity score model covariates** | | | | |
| Age, years | 0.231 | 0.189 | 0.008 | 0.000 |
| ECOG PS | 0.053 | 0.064 | 0.017 | 0.009 |
| Current treatment line | 0.429 | 0.181 | 0.026 | 0.021 |
| Initial FIGO stage | 0.057 | 0.280 | 0.022 | 0.000 |
| Disease status | 0.031 | 0.075 | 0.017 | 0.000 |
| **Additional clinically relevant baseline covariates** | | | | |
| BMI, kg/m² | 0.086 | 0.146 | 0.118 | 0.159 |
| Menopausal status | 0.071 | 0.059 | 0.014 | 0.019 |
| Histology | 0.046 | 0.000 | 0.084 | 0.025 |
| Tumor grade | 0.025 | 0.209 | 0.041 | 0.061 |
| Recurrence interval, months | 0.199 | 0.251 | 0.218 | 0.226 |
| Prior pelvic radiotherapy | 0.041 | 0.061 | 0.096 | 0.069 |
| Recurrence field | 0.056 | 0.134 | 0.096 | 0.069 |
| Distant metastasis | 0.142 | 0.192 | 0.149 | 0.136 |
| Metastatic site count | 0.142 | 0.293 | 0.210 | 0.162 |
| Measurable disease | 0.010 | 0.000 | 0.056 | 0.079 |
| Lung metastasis | 0.069 | 0.120 | 0.086 | 0.064 |
| Liver metastasis | 0.057 | 0.381 | 0.052 | 0.055 |
| Bone metastasis | 0.036 | 0.000 | 0.041 | 0.073 |
| Lymph-node-only disease | 0.005 | 0.263 | 0.109 | 0.100 |
| Prior cervical cancer surgery | 0.052 | 0.241 | 0.008 | 0.009 |
| Prior platinum therapy | 0.142 | 0.068 | 0.188 | 0.160 |
| Prior bevacizumab | 0.164 | 0.080 | 0.106 | 0.126 |
| Prior PD-1/PD-L1 therapy | 0.443 | 0.582 | 0.401 | 0.432 |
| SCC-Ag, ng/mL | 0.184 | 0.483 | 0.154 | 0.158 |
| Hb, g/L | 0.508 | 0.550 | 0.426 | 0.397 |
| Alb, g/L | 0.074 | 0.076 | 0.036 | 0.026 |
| LDH, U/L | 0.136 | 0.117 | 0.151 | 0.170 |
| NLR | 0.224 | 0.296 | 0.168 | 0.180 |
| PLR | 0.384 | 0.435 | 0.355 | 0.397 |
| PNI | 0.177 | 0.207 | 0.164 | 0.168 |
| PD-L1 CPS | 0.065 | 0.408 | 0.094 | 0.088 |
| **Summary diagnostics** | | | | |
| Analytic n or effective sample size | 111 | 68 | 103.1 | 96.9 |
| PSM matched pairs |  | 34 |  |  |
| Maximum absolute SMD | 0.508 | 0.582 | 0.426 | 0.432 |
| Covariates with SMD >0.10, n | 14 | 21 | 14 | 13 |

**Note:** Values in covariate rows are absolute standardized mean differences. The reduced propensity score model included age, ECOG PS, current treatment line, initial FIGO stage, and disease status. Additional clinically relevant baseline covariates were reported descriptively. Residual imbalance was evaluated using the full-cohort baseline-adjusted primary model and residual imbalance-adjusted sensitivity analyses.

**Table S2. Model Assumptions and Numerical Stability Diagnostics**

| **Diagnostic domain** | **Diagnostic item** | **Method** | **Statistic** | ***P* value** | **Conclusion** |
| --- | --- | --- | --- | --- | --- |
| **Cox model assumptions** | | | | | |
| Cox model assumptions | PFS baseline-adjusted Cox model | Global Schoenfeld residual PH test | chi-square=4.68 | 0.586 | pass |
| Cox model assumptions | PFS treatment effect | Schoenfeld residual PH test | chi-square=0.19 | 0.662 | pass |
| Cox model assumptions | PFS liver metastasis | Schoenfeld residual PH test | chi-square=0.38 | 0.538 | pass |
| Cox model assumptions | PFS baseline log1p NLR | Schoenfeld residual PH test | chi-square=0.41 | 0.521 | pass |
| Cox model assumptions | PFS recurrence field | Schoenfeld residual PH test | chi-square=2.94 | 0.401 | pass |
| Cox model assumptions | OS Cox model, secondary endpoint | Global Schoenfeld residual PH test | chi-square=0.14 | 0.708 | acceptable |
| Cox model assumptions | OS treatment effect, secondary endpoint | Schoenfeld residual PH test | chi-square=0.14 | 0.708 | acceptable |
| **Influence and collinearity** | | | | | |
| Influence and collinearity | PFS main model complexity | Events per model coefficient | 68 events / 6 df = 11.3 |  | pass |
| Influence and collinearity | Residual imbalance-adjusted model complexity | Events per model coefficient | 68 events / 8 df = 8.5 |  | acceptable |
| Influence and collinearity | PFS main model influence | Maximum absolute dfbeta | 0.180 |  | pass |
| Influence and collinearity | PFS main model residuals | Maximum absolute deviance residual | 2.62 |  | pass |
| Influence and collinearity | PFS main model collinearity | Maximum VIF across model matrix columns | 1.323 |  | pass |
| Influence and collinearity | PFS main model design correlation | Maximum absolute pairwise correlation | 0.294 |  | pass |
| **Propensity score and weighting diagnostics** | | | | | |
| Propensity score and weighting diagnostics | Reduced PS model coefficients | Finite coefficient check | max \|beta\|=3.36 |  | pass |
| Propensity score and weighting diagnostics | Propensity score range | Reduced PS model | study 0.203-0.751; control 0.206-0.661 |  | acceptable |
| Propensity score and weighting diagnostics | Common support coverage | Empirical common support | overall 96.4%; study 90.5%; control 100.0% |  | acceptable |
| Propensity score and weighting diagnostics | Extreme propensity scores | PS <0.05 or >0.95 | 0 |  | pass |
| Propensity score and weighting diagnostics | Raw stabilized IPTW range | Stabilized IPTW | 0.504-1.864 |  | acceptable |
| Propensity score and weighting diagnostics | Trimmed IPTW range | 1st-99th percentile truncation | 0.567-1.830 |  | acceptable |
| Propensity score and weighting diagnostics | Trimmed IPTW ESS | Effective sample size | 103.1 |  | acceptable |
| Propensity score and weighting diagnostics | Overlap weight range | Overlap weighting | 0.206-0.797 |  | acceptable |
| Propensity score and weighting diagnostics | Overlap weighting ESS | Effective sample size | 96.9 |  | acceptable |
| Propensity score and weighting diagnostics | PSM retained sample | Nearest-neighbor 1:1 PSM | 34 pairs; *n*=68 |  | acceptable |
| Propensity score and weighting diagnostics | Reduced PS covariate balance after PSM | Maximum absolute SMD | 0.280 |  | caution |
| Propensity score and weighting diagnostics | Reduced PS covariate balance after trimmed IPTW | Maximum absolute SMD | 0.026 |  | pass |
| Propensity score and weighting diagnostics | Reduced PS covariate balance after overlap weighting | Maximum absolute SMD | 0.021 |  | pass |
| **Binary outcome numerical stability** | | | | | |
| Binary outcome numerical stability | ORR | 2x2 zero-cell and finite logistic coefficient check | minimum cell=20; max \|beta\|=0.72 |  | pass |
| Binary outcome numerical stability | DCR | 2x2 zero-cell and finite logistic coefficient check | minimum cell=6; max \|beta\|=1.03 |  | pass |
| Binary outcome numerical stability | Grade 3-5 TRAE | 2x2 zero-cell and finite logistic coefficient check | minimum cell=8; max \|beta\|=0.94 |  | pass |
| Binary outcome numerical stability | Grade 3-5 myelosuppression | 2x2 zero-cell and finite logistic coefficient check | minimum cell=4; max \|beta\|=1.49 |  | pass |
| Binary outcome numerical stability | Hypertension | 2x2 zero-cell and finite logistic coefficient check | minimum cell=12; max \|beta\|=1.56 |  | pass |
| Binary outcome numerical stability | Proteinuria | 2x2 zero-cell and finite logistic coefficient check | minimum cell=8; max \|beta\|=2.03 |  | pass |
| Binary outcome numerical stability | Thyroid dysfunction | 2x2 zero-cell and finite logistic coefficient check | minimum cell=8; max \|beta\|=1.77 |  | pass |
| Binary outcome numerical stability | Dose modification or interruption | 2x2 zero-cell and finite logistic coefficient check | minimum cell=14; max \|beta\|=1.04 |  | pass |
| Binary outcome numerical stability | Treatment discontinuation | 2x2 zero-cell and finite logistic coefficient check | minimum cell=20; max \|beta\|=0.69 |  | pass |

**Note:** The diagnostics were restricted to the finalized models and propensity score strategy. The PFS baseline-adjusted Cox model included treatment group, liver metastasis, log1p-transformed NLR, and recurrence field. The residual imbalance-adjusted sensitivity model additionally included Hb and prior PD-1/PD-L1 exposure. The reduced propensity score model included age, ECOG PS, current treatment line, initial FIGO stage, and disease status. Safety and response endpoints were checked for zero cells and finite logistic coefficients; exact methods were used where appropriate. Conclusions were labeled as pass, acceptable, or caution.
